# Increased risk of reoperation and failure to attain clinically relevant improvement following autologous chondrocyte implantation of the knee in female patients and individuals with previous surgeries: a time-to-event analysis based on the German cartilage registry (KnorpelRegister DGOU)

**DOI:** 10.1007/s00167-023-07615-5

**Published:** 2023-11-11

**Authors:** Alexander Bumberger, Marco Christopher Rupp, Christian Lattermann, Anne Kleiner, Philipp Niemeyer

**Affiliations:** 1 Department of Orthopedics and Trauma Surgery, Medical University of Vienna, AKH Wien, Waehringer Guertel 18-20, 1090 Vienna, Austria; 2 OCM, Munich, Germany; 3 The Steadman Philippon Research Institute, 181 West Meadows Drive, Suite 400, Vail, CO USA; 4 Department of Orthopaedic Sports Medicine, Hospital Rechts Der Isar, Technical University of Munich, Munich, Germany; 5 Brigham and Women’s Hospital, Harvard Medical School, Boston, MA USA; 6 Department of Orthopedics and Trauma Surgery, University Medical Center Freiburg, Albert-Ludwig University of Freiburg, Freiburg, Germany

**Keywords:** Knee, Joint, Articular, Cartilage, Regeneration, Restoration, Survival, Analysis, Failure, Complication, Revision, ACI, Autologous chondrocyte implantation, Autologous chondrocyte transplantation, Registry

## Abstract

**Purpose:**

This study aimed to analyze the risk of reoperation following autologous chondrocyte implantation (ACI) of the knee utilizing third-generation ACI products in a time-to-event analysis and report on the associated patient-reported outcome measures (PROM) in case of reoperation.

**Methods:**

Patients undergoing ACI were included from a longitudinal database. Patient age, sex, body mass index (BMI), number of previous surgeries, lesion localization, lesion size, symptom duration, as well as time and type of reoperation was extracted. A cox proportional-hazards model was applied to investigate the influence of baseline variables on risk of reoperation. Reoperation was defined as any type of subsequent ipsilateral knee surgery, excluding hardware removal. The Knee Injury and Osteoarthritis Outcome Score (KOOS) was utilized to compare PROM between patients with and without reoperation.

**Results:**

A total of 2039 patients were included with 1359 (66.7%) having a minimum follow-up of 24 months. There were overall 243 reoperations (prevalence 17.9%). Minor arthroscopic procedures (*n* = 96, 39.5%) and revision cartilage repair procedures (*n* = 78, 32.1%) were the most common reoperations. Nineteen patients (0.9%) reported conversion arthroplasty at 17.7 (10.4) months after ACI. Female sex (HR 1.5, 95% CI [1.2, 2.0], *p* = 0.002) and the presence of 1–2 previous surgeries (HR 1.5, 95% CI [1.1, 2.0], *p* = 0.010), or more than 2 previous surgeries (HR 1.9, 95% CI [1.2, 2.9], *p* = 0.004) were significantly associated with increased risk of reoperation following ACI. Significantly less patients surpassed the minimal clinically important difference (MCID) in the reoperation group at 24 months regarding the KOOS subscores pain (OR 1.6, 95% CI [1.1, 2.2]), quality of life (OR 2.2, 95% CI [1.6, 3.2]), symptoms (OR 2.0 [1.4, 2.9]), and sports (OR 2.0 [1.4, 2.8]).

**Conclusion:**

Female patients and individuals with a history of previous surgeries face an elevated risk of requiring reoperation after undergoing ACI, which is associated with failure to attain clinically relevant improvements. A thorough evaluation of the indications for ACI is paramount, particularly when patients have a history of previous surgeries.

**Level of evidence:**

Level III.

**Supplementary Information:**

The online version contains supplementary material available at 10.1007/s00167-023-07615-5.

## Introduction

Autologous chondrocyte implantation (ACI) has undergone continuous refinement since its initial clinical application in knee joints by Brittberg et al. [4]. Multiple randomized-controlled trials (RCT) have unequivocally established its potential for regenerating focal lesions of articular cartilage in the knee and its superiority over microfracture (MF) regarding patient-reported outcome measures (PROM) and tissue quality [3, 5, 12, 22, 23]. Nonetheless, evaluating the efficacy of surgical interventions requires assessing the need for reoperation as a critical metric. Emerging evidence suggests that specific patient-related factors may increase the risk of reoperation following ACI [1, 7, 10, 11, 13, 16, 17, 19, 20, 26].

In 2012, Jungmann et al. highlighted that female sex, a higher number of previous surgeries, previous bone-marrow stimulation (BMS) and the use of a periosteal patch (associated with first-generation ACI) were linked to an elevated risk of reoperation following ACI for focal cartilage lesions of the knee [13]. Notably, contemporary matrix-associated (third generation) ACI products have eliminated the need for periosteal patches or chondrogenic membranes [2], while previous BMS is an ongoing concern [24]. Anigwe et al. [1] recently identified higher age and tobacco use as significant predictors of an increased risk of conversion to arthroplasty following ACI, and female sex to be associated with a generally higher reoperation risk.

Studies examining the clinical significance of these reoperations remain limited. Ogura et al. observed a trend toward improved graft survival in patients undergoing revision ACI without prior cartilage regenerative (CR) procedures before the initial ACI [19]. This improvement correlated with pain relief, enhanced function, and high patient satisfaction. However, their study was based on a relatively small cohort of 53 patients, potentially lacking the statistical power to detect significant differences in graft survival.

Therefore, the primary objective of this study was to conduct a comprehensive analysis of the risk of reoperation following ACI of the knee using third-generation ACI products. This analysis employed a time-to-event framework and concurrently assessed associated patient-reported outcome measures (PROM) in cases requiring reoperation. We hypothesized that certain patient- and lesion-specific parameters influence the risk of reoperation following ACI and correlate with postoperative PROM.

## Materials and methods

### Data source

Data for this study were sourced from the German Cartilage Registry (KnorpelRegister DGOU). Ethical approval for this study was obtained from the Ethic﻿s Commission of the Medical Center, University of Freiburg (Approval number: EK-FR 105/13_130795). The registry is conducted in strict adherence to the Declaration of Helsinki and is registered at germanctr.de (DRKS00005617). Informed written consent is obtained at the time of cartilage biopsy. Subsequently, patients are automatically contacted via email to complete a PROM questionnaire and report potential reoperations through free-text entries at 6, 12, 24, 36 and 60 months postoperatively.

### Study cohort

**Fig. 1:**
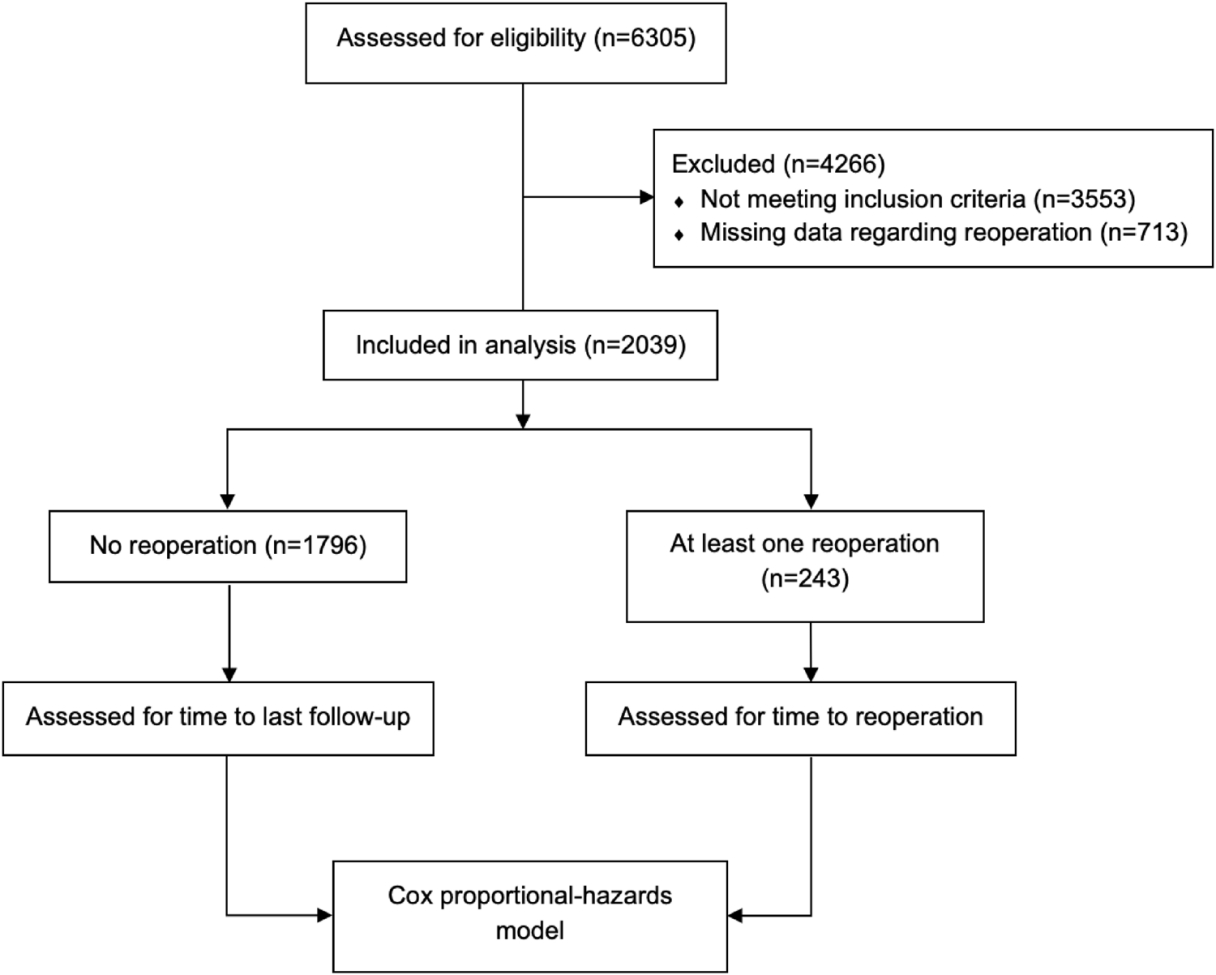
CONSORT Flow Diagram showing patient inclusion from the German Cartilage Registry (KnorpelRegister DGOU)

A registry-based time-to-event analysis was performed, including consecutive patients who underwent third-generation ACI, with or without concomitant procedures, for focal cartilage lesions of the knee. The study cohort had a minimum follow-up duration of 6 months, extending up to 5 years. Patients with missing data regarding the incidence of reoperation were excluded (Fig. 1).

### Data collection

Patient demographic and clinical data were collected, including age, sex, body mass index (BMI), number of previous surgeries, lesion localization, lesion size, symptom duration, and the date and type of reoperation. The type of reoperation was meticulously reviewed and categorized by a fellowship-trained orthopedic surgeon (PN). Cases with unlcear descriptions were categorized as “not specified”. Hardware removal, such as after concomitant osteotomy, was not considered a reoperation.

### Categorization of reoperation procedures

A protocol for categorizing reoperation procedures was developed based on consensus between two orthopedic surgeons (MCR, AB). Any disagreements were resolved through discussion until consensus was reached. Reoperation procedures were categorized as follows:

- Non-reconstructive arthroscopic procedures (e.g., lysis of adhesions, meniscectomy, etc.)
- Cartilage revision procedures (e.g., chondroplasty, ACI, microfracture, etc.)
- Conversion to arthroplasty (e.g., UKA, PFA, TKA)
- Realignment procedures (e.g., osteotomy, tibial tubercle osteotomy)
- Ligament reconstruction/repair procedures (e.g., ACL reconstruction, MPFL reconstruction)
- Other reoperations (including various procedures such as joint infection treatment, wound-healing disorders, fracture management, etc.)

### Outcome measures

The primary outcome measure was the occurance of any reoperation. Secondary outcome measures included conversion to arthroplasty and the assessment of Knee Injury and Osteoarthritis Outcome Score (KOOS) at baseline, 6, 12, 24 and 36 months following ACI. Additionally, KOOS subscores were analyzed with regard to the validated minimal clinically important difference (MCID) at 24 months following ACI [18].

### Analysis of previous surgeries

To assess the significance of previous surgeries, the exact type of previous surgeries was extracted and an inter-group comparisons between patients with and without reoperation following ACI was performed.

### Statistical analysis

#### Cox proportional-hazards analysis

A cox proportional-hazards analysis was performed, based on the status of reoperation (no reoperation vs. at least one reoperation) observed during the study period. Independent parameters included patient age, sex, body mass index (BMI), symptom duration, lesion localization, lesion size and the number of previous surgeries. These same parameters were used in the sub-analysis for secondary outcome measures.

The proportional-hazards assumption was assessed visually through Schoenfeld residuals vs. time plots for continuous variables and using log-minus-log (LML) survival plots for categorical variables. This analysis confirmed the proportional-hazards assumption for all independent variables included. Parameter covariance was assessed and visualized in a correlation matrix heatmap. Furthermore, the included predictor variables were assessed for multicollinearity, quantifying how well each variable could be predicted from the others, thus assessing parameter independence, and was expressed as *R*^*2*^. The overall model fit was assessed using Harrell’s C-statistic.

#### Data presentation

Continuous variables were reported as mean ± SD, while categorical variables were presented as counts and percentages. Absolute numbers and percentages were reported with one decimal place, while *p* values were presented with three decimal places. To compare continuous variables between groups, *t* tests for independent samples were used. Categorical variables were compared using Pearson’s chi-square test or Fisher’s Exact Test. Levene’s test was applied to assess homogeneity of variances for continuous variables, and in case of statistical significance, Welch’s test was conducted, with *p* values reported as necessary. The normality of data distribution was confirmed using the Shapiro–Wilk Test.

#### Statistical significance

Statistical significance was set at a *p* value of < 0.05. To account for accumulation of alpha-errors in inter-group comparisons of previous surgeries between patients with and without reoperations, the significance level was adjusted to 0.004 using Bonferroni’s correction.

#### Statistical software

All statistical analyses were performed using Prism 9 (GraphPad Software).

#### Review of statistical methods

The statistical methods were meticulously reviewed in accordance with a recent publication by Pruneski et al. on survival analyses in orthopedics [21].

## Results

### Descriptive analysis

A total of 2039 patients met the inclusion criteria (Table 1). The mean follow-up duration was 25.9 ± 17.9 months, ranging from 6 to 63 months. Of these patients, 1359 (66.7%) had a minimum follow-up of 24 months, with an overall reoperation rate of 17.9% (243/1359). Among these, 17 patients (0.8%) reported two distinct reoperations. The mean time to the first reoperation was 15.6 ± 12.3 months. Arthroscopic procedures (39.5%) and revision cartilage repair procedures (32.1%) were the most frequently reported types of reoperations (Table 2).

**Table 1:** Patient baseline characteristics

		No reoperation (*n* = 1796)		Reoperation (*n* = 243)		*P* value
		Mean ± SD	*N* (%)	Mean ± SD	*N* (%)	
Age		35.3 ± 10.6		37.1 ± 10.1		0.012*
Sex	Male		1077 ± 60.2		125 ± 50.6	0.004*
	Female		711 ± 39.8		122 ± 49.2	
BMI		26.3 ± 4.3		26.0 ± 4.9		n.s
Symptom duration [months]		22.7 ± 35.2		27.4 ± 43.1		n.s
Lesion localization	PF		997 ± 56.2		119 ± 48.8	n.s
	TF		776 ± 43.8		125 ± 51.2	
Lesions size [cm^2^]		4.2 ± 2.2		4.2 ± 2.1		n.s
Previous surgeries	0		785 ± 43.8		84 ± 34.0	0.001*
	1—2		870 ± 48.6		131 ± 53.0	
	> 2		136 ± 7.6		32 ± 13.2	

Baseline characteristics of patients reporting either no- or at least one reoperation following *ACI* autologous chondrocyte implantation, *BMI* body mass index, *PF* patello-femoral, *TF* tibio-femoral

*p < .05

**Table 2:** Reoperations following autologous chondrocyte implantation (ACI)

	*N*	Percentage of reoperations (*n* = 270)	Percentage of cases (*n* = 2039)
**Non-reconstructive arthroscopic procedures**	**96**	**39.5**	**4.7**
Lysis of adhesions	51	21.0	2.5
Meniscectomy	24	9.9	1.2
Arthroscopy (not specified)	16	6.6	0.8
Loose body removal	3	1.2	0.1
Cyclops lesion	2	0.8	0.1
**Cartilage revision procedures**	**78**	**32.1**	**3.8**
Chondroplasty	26	10.7	1.3
Cartilage (not specified)	18	7.4	0.9
ACI	15	6.2	0.7
BMS	14	5.8	0.7
OAT	3	1.2	0.1
mBMS	2	0.8	0.1
**Conversion to arthroplasty**	**20**	**8.2**	**1.0**
Arthroplasty (UKA)	10	4.1	0.5
Arthroplasty (PFJ)	6	2.5	0.3
Arthroplasty (TKA)	4	1.6	0.2
**Realignment procedures**	**14**	**5.8**	**0.7**
Osteotomy	12	4.9	0.6
Patella realignment (not specified)	2	0.8	0.1
**Ligament reconstruction/repair procedures**	**14**	**5.8**	**0.7**
ACL reconstruction	9	3.7	0.4
MPFL reconstruction	4	1.6	0.2
MCL-refixation	1	0.4	0.0
**Other reoperations**	**48**	**19.8**	**2.4**
Not specified	21	8.6	1.0
Joint infection	7	2.9	0.3
Surgical site infection	4	1.6	0.2
Wound healing disorder	3	1.2	0.1
Scar revision	2	0.8	0.1
Screw loosening	2	0.8	0.1
Hematoma evacuation	1	0.4	0.0
Patella fracture	1	0.4	0.0
Tibia fracture	1	0.4	0.0
Lateral retinaculum release	1	0.4	0.0
Screw fracture	1	0.4	0.0
Arterial injury	1	0.4	0.0
Tendon repair (not specified)	1	0.4	0.0
Neuroma	1	0.4	0.0
Osteophyte removal	1	0.4	0.0
**Total**	**270**	**100.0**	**13.2**

The bold text represents the sub-categories

Total number of reoperations reported. 10 patients (0.5%) reported two aspects of the first reoperation. 17 patients (0.8%) reported 2 distinct reoperations

*ACI* autologous chondrocyte implantation, *ACL* anterior cruciate ligament, *BMS* bone marrow stimulation, *mBMS* matrix-associated BMS, *MPFL* medial patello-femoral ligament, *MCL* medial collateral ligament, *OAT* osteochondral autograft transplantation

### Time-to-event analyses

#### Cox proportional-hazards model for reoperation

**Fig. 2:**
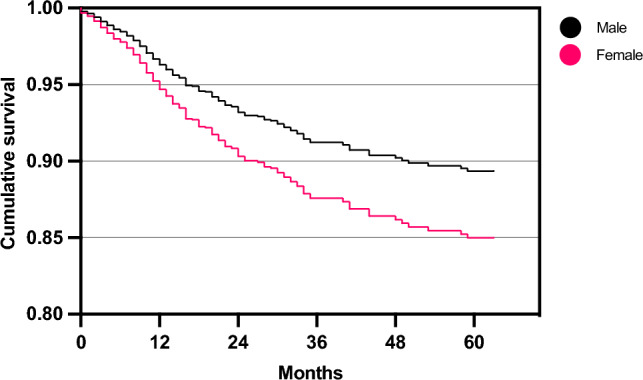
Estimated survival plot for male and female patients, based on the cox proportional-hazards model for risk of reoperation following autologous chondrocyte implantation (ACI). Female patients were at a higher risk of reoperation compared to male patients (HR 1.52, 95% CI [1.16, 1.99], *p* = 0.002)

**Fig. 3:**
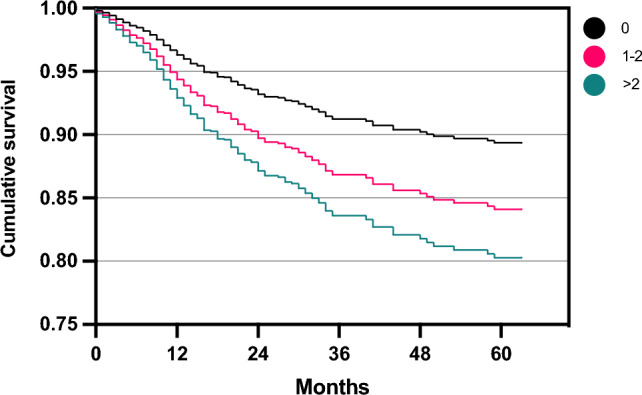
Estimated survival plot for patients with either no (0), 1–2, or > 2 previous surgeries, based on the cox proportional-hazards model for risk of reoperation following autologous chondrocyte implantation (ACI). Patients with 1–2 (HR 1.47, 95% CI [1.10, 1.99], *p* = 0.010) and > 2 previous surgeries (HR 1.91, 95% CI [1.21, 2.91], *p* = 0.004) were at a higher risk of reoperation compared to patients without previous surgeries

**Fig. 4:**
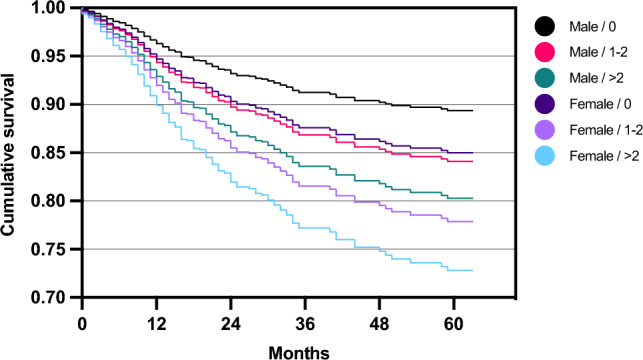
Estimated survival plot for combined effects of patient sex and number of previous surgeries, based on the cox proportional-hazards model for risk of reoperation following autologous chondrocyte implantation (ACI). Male patients without previous surgeries showed the lowest risk, female patients with > 2 previous surgeries the highest risk of undergoing reoperation. However, these findings were statistically not significant

**Fig. 5:**
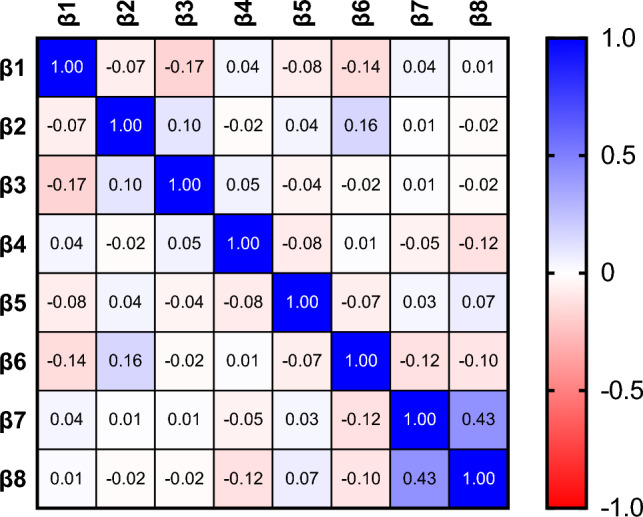
Correlation matrix heatmap demonstrating parameter covariance of included predictors in the cox proportional-hazards model regarding risk of reoperation following autologous chondrocyte implantation (ACI). *β1* = age, *β2* = sex, *β3* = body mass index, *β4* = symptom duration, *β5* = defect size, *β6* = defect localization (tibio-femoral), *β7* = previous surgeries (1–2), *β8* = previous surgeries (> 2). There was a moderate covariance between the two levels of previous surgeries, but otherwise weak inter-parameter covariance

The cox proportional-hazards model revealed significant associations between predictor variables and variations in hazards rates for reoperation (*R*^2^ = 0.59, 95% CI [0.56, 0.63], *p* < 0.001). Specifically, female sex (HR 1.5, 95% CI [1.2, 2.0], *p* = 0.002) and the presence of 1–2 previous surgeries (HR 1.5, 95% CI [1.1, 2.0], *p* = 0.010) or more than 2 previous surgeries (HR 1.9, 95% CI [1.2, 2.9], *p* = 0.004) were significantly associated with an increased risk of reoperation (Figs. 2,  3, Table 3). The estimated combined effects of sex and previous surgeries are shown in Fig. 4. However, none of these combined effects were statistically significant. Independence of predictor variables was assessed as outlined in the methods section with coefficients of multicollinearity shown in Supplementary Table 1 and coefficients of correlation visualized in Fig. 5. Patients reporting reoperation had a significantly higher prevalence of previous partial meniscectomy (23.1% vs. 14.9%, *p* < 0.001). No other significant differences were observed (Table 4).

**Table 3:** Cox proportional-hazards model for reoperation

	HR (estimate)	95% CI	*P* value
Age	1.0	[1.0, 1.0]	n.s
Sex (female)	1.5	[1.2, 2.0]	0.002*
BMI	1.0	[1.0, 1.0]	n.s
Symptom duration	1.0	[1.0, 1.0]	n.s
Lesion localization (TF)	1.2	[0.9, 1.6]	n.s
Lesion size	1.0	[1.0, 1.0]	n.s
Previous surgeries (1–2)	1.5	[1.1, 2.0]	0.010*
Previous surgeries (> 2)	1.9	[1.2, 2.9]	0.004*

Hazard ratios (HR) for predictor variables included in the cox proportional-hazards model for risk of reoperation following autologous chondrocyte implantation (ACI)

*BMI* body mass index, *TF* tibio-femoral

*p < 0.05

**Table 4:** Surgeries prior to autologous chondrocyte implantation (ACI)

	No reoperation		Reoperation		*P* value
	*N*	%	*N*	%	
Meniscectomy	267	14.9	57	23.1	< 0.001*
Cartilage repair (same localization)	264	14.7	49	19.8	n.s
Not specified	232	12.9	37	15.0	n.s
ACL reconstruction	191	10.7	27	10.9	n.s
MPFL reconstruction	89	5.0	13	5.3	n.s
Meniscus repair	57	3.2	8	3.2	n.s
Cartilage repair (different localization)	49	2.7	5	2.0	n.s
Osteotomy	34	1.9	5	2.0	n.s
Fracture surgery	16	0.9	2	0.8	n.s
Patella realigment	13	0.7	3	1.2	n.s
Collateral ligament surgery	5	0.3	1	0.4	n.s
PCL reconstruction	2	0.1	0	0.0	n.s

Surgeries prior to initial autologous chondrocyte implantation (ACI) for patients with and without reoperation shown in absolute numbers and intra-group percentage

*ACL* anterior cruciate ligament, *MPFL* medial patello-femoral ligament, *PCL* posterior cruciate ligament

Adjusted level of significance = 0.004

*p < 0.004

#### Cox proportional-hazards model for conversion arthroplasty

The cox proportional-hazards model demonstrated significant associations between predictor variables and variations in hazard rates for conversion arthroplasty (*R*^*2*^ = 0.810, 95% CI [0.704, 0.916], *p* < 0.001). In total, 19 patients (0.9%) reported having undergone unicondylar (UKA) or total knee arthroplasty (TKA) following ACI. These patients were significantly older, with an average of 42.8 ± 8.3 years, compared to other patients with an average age of 35.4 ± 10.5 years (*p* = 0.003). No further inter-group differences regarding baseline characteristics were observed. The mean time to conversion arthroplasty was 17.6 ± 10.4 months from ACI. Cox-regression revealed that higher patient age (HR 1.1, 95% CI [1.0, 1.1], *p* = 0.010), female sex (HR 3.3, 95% CI [1.2, 10.7], *p* = 0.027) and having undergone more than 2 previous surgeries (HR 5.6, 95% CI [1.4, 23.1], *p* = 0.012) were significantly associated with an increased risk of conversion arthroplasty (Table 5).

**Table 5:** Cox proportional-hazards model for conversion arthroplasty

	HR (estimate)	95% CI	*P* value
Age	1.1	[1.0, 1.1]	0.01*
Sex (female)	3.3	[1.2, 10.7]	0.027*
BMI	1.1	[1.0, 1.2]	n.s
Symptom duration	1.0	[1.0, 1.0]	n.s
Lesion localization (TF)	2.3	[0.8, 7.4]	n.s
Lesion size	1.0	[1.0, 1.0]	n.s
Previous surgeries (1–2)	1.7	[0.5, 6.6]	n.s
Previous surgeries (> 2)	5.6	[1.4, 23.1]	0.012*

Hazard ratios (HR) for predictor variables included in the cox proportional-hazards model for risk of conversion arthroplasty following autologous chondrocyte implantation

*BMI* body mass index, *TF* tibio-femoral

*p < 0.05

### Patient-reported outcomes in case of reoperation

**Fig. 6:**
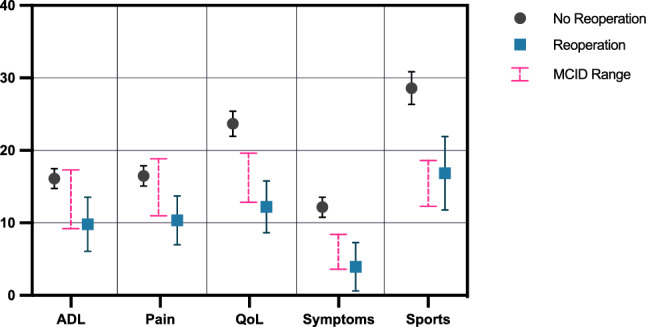
Average group-level Knee Injury and Osteoarthritis Outcome Score (KOOS) subscores of patients with and without reoperation following autologous chondrocyte implantation (ACI). Minimal clinically important difference (MCID) range as published by Ogura et al. [17]. On average group-level, patients with reoperations surpassed the MCID in Activities of Daily Living (ADL), Symptoms and Sports, but failed to surpass the MCID in Pain and Quality of Life (QoL) subscores

Patients undergoing reoperation exhibited significantly worse overall KOOS at all follow-ups (Table 6), although they still surpassed the MCID in activities of daily living (ADL, 9.8 ± 22.6), symptoms (4.0 ± 20.3) and sports/recreational activities (16.8 ± 29.5) at the group level, 24 months postoperatively (Table 7, Fig. 6). The percentage of individual patients surpassing the MCID was significantly lower in the reoperation group for all subscores, except ADL (Table 8).

**Table 6:** Overall KOOS

	No reoperation		Reoperation		*P* value
	Mean	± SD	Mean	± SD	
KOOS Preop	59.5	± 17.4	53.0	± 17.0	< 0.001*
KOOS–6 M	70.6	± 15.5	59.7	± 17.4	< 0.001*
KOOS–12 M	75.6	± 15.5	59.7	± 18.3	< 0.001*
KOOS–24 M	76.9	± 16.2	62.2	± 19.3	< 0.001*
KOOS–36 M	77.7	± 16.8	64.2	± 20.1	< 0.001*

Comparison of the overall Knee Injury and Osteoarthritis Outcome Score (KOOS) between patients with and without reoperation following autologous chondrocyte implantation (ACI) at different follow-ups. 6, 12, 24 and 36 months (6 M, 12 M, 24 M, 36 M)

*p < 0.05

**Table 7:** Improvement of KOOS subscores at 24 months follow-up

	No reoperation		Reoperation		*P* value
	Mean	± SD	Mean	± SD	
KOOS–ADL	16.1	± 20.0	9.8	± 22.6	< 0.001*
KOOS–Pain	16.5	± 20.6	10.4	± 20.4	< 0.001*
KOOS–QoL	23.7	± 25.3	12.2	± 21.6	< 0.001*
KOOS–Symptoms	12.2	± 20.3	4.0	± 20.3	< 0.001*
KOOS–Sports	28.6	± 31.9	16.8	± 29.5	< 0.001*

Comparison of Knee Injury and Osteoarthritis Outcome Score (KOOS) subscores at 24 months between patients with and without reoperation

*ADL* activities of daily living, *QoL* quality of life

*p < 0.05

**Table 8:** Percentage of patients surpassing MCID of KOOS at 24 months follow-up

	No reoperation		Reoperation		OR	*P* value
	*N*	%	*N*	%		
KOOS–ADL	492	59.7	76	52.8		n.s
KOOS–Pain	509	61.8	73	50.7	1.6 [1.1, 2.2]	0.013*
KOOS–QoL	515	62.8	62	43.1	2.2 [1.6, 3.2]	< 0.001*
KOOS–Symptoms	538	65.4	70	48.6	2.0 [1.4, 2.9]	< 0.001*
KOOS–Sports	536	69.2	71	53.4	2.0 [1.4, 2.8]	< 0.001*

Number of patients surpassing the minimal clinically important difference (*MCID*) of Knee Injury and Osteoarthritis Outcome Score (*KOOS*) subscores at 24 months following autologous chondrocyte implantation (*ACI*). *ADL* activities of daily living, *QoL* quality of life

*p < 0.05

## Discussion

The primary finding of this study was the significant association of previous surgeries and female sex with an increased risk of reoperation following ACI for focal articular cartilage lesions of the knee joint. Patients with more than two previous surgeries had nearly twice the risk of undergoing reoperation following ACI, while female patients had roughly a 1.5-fold increased risk compared to male patients. Additionally, reoperation was associated with significantly worse KOOS at all follow-ups, including baseline. Furthermore, these patients were less likely to surpass the MCID at the 24-month follow-up.

The overall prevalence of reoperations (patients with at least one reoperation) in our cohort was 17.9%, which is lower than the recently reported 30.4% by Anigwe et al. and may be attributable to the shorter follow-up period in our study (2.2 ± 1.5 vs. 4.8 ± 3.3 years in Anigwe et al.), and the exclusive use of third-generation ACI in our cohort [1]. Anigwe et al. also demonstrated a significant decrease of reoperations for ACI performed after 2017 [1]. Harris et al. reported a similar reoperation rate of 33% in a systematic review, primarily including patients who underwent first-generation ACI [11]. Niethammer et al. reported a revision rate of 20.4% in a series of third-generation ACI with a minimum follow-up of 2 years [17], consistent with our study, although we reported all reoperations rather than revisions. Conversely, a systematic review from 2016 reported lower reoperation rates compared to our findings, with a pooled rate of 5% for patients undergoing reoperation at 2–5 years follow-up [26]. Furthermore, a recently published 9-year follow-up study of patients undergoing ACI reported a very low revision surgery rate of 7% [6]. This variability in reoperation rates underscores the need for further investigation. While we acknowledge the potential for an attrition bias due to a higher loss to follow-up in patients undergoing reoperations, our findings are consistent with previous studies [6, 11, 16, 20, 26].

The most frequently performed types of reoperations in our study were lysis of adhesions, chondroplasty, and meniscectomy. These findings are similar to those reported by Anigwe et al., who identified chondroplasty, meniscectomy and microfracture among the top three reoperation types [1]. Harris et al. also reported lysis of adhesions among the most frequently performed reoperations for arthrotomy-based ACI, along with chondroplasty (“graft debridement”) and manipulation under anesthesia [11]. However, it is worth noting that lysis of adhesions was predominantly reported in cohorts undergoing first-generation ACI. In our study, “lysis of adhesions” does not necessarily imply a distinct diagnosis of arthrofibrosis but could also refer to an arthroscopic debridement of minor scar tissue or partial synovectomy in case of persisting pain or mechanical symptoms following ACI.

The unfavorable effect of female sex on cartilage repair outcomes has been consistently documented in the literature [1, 8–10, 13–15]. Our findings align with these reports. Jungmann et al. reported an overall odds ratio of 1.7 for female patients regarding reoperation following ACI [13]. Filardo et al. observed worse clinical outcomes in female patients, although this effect was attenuated in a matched analysis considering different lesion patterns and causes of injury [9]. Kreuz et al. found the worst clinical outcomes in female patients with patellar lesions, despite men having, on average, larger patellar lesions [14]. The authors hypothesized that the inferior outcome scores in women might be attributable to insufficient proprioception and imbalances in muscle forces, and lower isokinetic strength, as demonstrated in an earlier study [15]. Faber et al. highlighted gender-specific discrepancies in terms of patient baseline characteristics, showing that women were older than men at the time of cartilage repair, had more previous surgeries and a longer symptom duration [8].

To elaborate on the interdependence of female sex and associated parameters, we conducted an analysis of covariance and assessed multicollinearity in our regression model. We found that the correlation coefficients between female sex and the other parameters were generally weak, with the highest coefficient observed between female sex and lesion localization (*r* = 0.16). Interestingly, the positive coefficient suggested that female sex was associated with a higher prevalence of tibio-femoral lesions, contrary to previous findings [8, 15]. The multicollinearity analysis showed an *R*^*2*^ value of 0.404 for female sex, indicating that 40.4% of the variability could be explained by the other variables in our model. In essence, this parameter displayed an intermediate association with the remaining variables. Given these findins and previous data, it is evident that female sex is typically associated with other baseline patient and lesion characteristics that are routinely recorded. However, our data also suggest that there may be additional gender-related differences yet to be investigated specifically.

The significant impact of previous surgeries on the risk of reoperation and worse KOOS following ACI aligns with existing literature [13, 25]. Furthermore, we demonstrated that this risk increases with the number of previous procedures. While the hazard ratio (HR) for 1–2 previous surgeries was approximately 1.5, having more than 2 previous procedures raised the HR to almost 2. These patients also failed to surpass the MCID of the KOOS subscores of pain and quality of life (QoL). Notably, a history of previous CR procedures is of particular concern, as reported by Seiferth et al. [25].

Our study also identified higher patient age, female sex, and a history of more than two previous surgeries as significantly associated with an increased risk of conversion to arthroplasty, consistent with the findings of Anigwe et al. [1]. The HR for patient age indicated a 7% increase in the risk of conversion arthroplasty per year, suggesting that a patient at 50 years had approximately twice the risk of a patient at 40 years for undergoing conversion arthroplasty following ACI. Female patients had over a threefold increased risk, while patients with more than 2 previous surgeries had an over fivefold increased risk of conversion to arthroplasty. The cox proportional-hazards model demonstrated a strong model fit for conversion to arthroplasty (*R*^*2*^ = 0.810, 95% CI [0.704, 0.916], *p* < 0.001), indicating that the risk of conversion arthroplasty could be accurately predicted based on patient age, sex, and history of previous surgeries.

In summary, this study, one of the largest reporting on reoperation rates and associated clinical findings following ACI, identified an increased risk of reoperation in female patients and patients with previous surgeries, which was linked to poorer clinical outcomes and a failure to surpass the MCID in most KOOS subscores. These findings have important implications for patient selection and managing patient expectations when scheduling ACI.

Several limitations should be acknowledged. The number of reoperations in our study may be subject to an attrition bias if there was a discrepancy in drop-out-rates between patients with and without reoperations. Additionally, as data on the type of reoperation were patient-reported via free-text entries, the exact type of reoperation was sometimes unclear and then categorized as “not specified”. Moreover, the patient-reported data carries the risk of underreporting. The cox proportional-hazards model for reoperation exhibited a weak model fit, suggesting limited accuracy in predicting reoperation risk based on the available parameters. Finally, our study included patients undergoing different types of CR and lesion localizations (tibio-femoral and patello-femoral), which should be considered when interpreting the data.

Reoperation and the failure to attain clinically relevant improvement following ACI of the knee are frustrating for both patients and physicians as the procedure requires two surgeries and an extensive rehabilitation protocol. Consequently, identifying patients at risk of reoperation and unfavorable clinical outcomes is paramount. The findings of the present study aid in streamlining this identification process by providing valuable insights into the implications of various baseline parameters.

## Conclusion

Female patients and individuals with a history of previous surgeries face an elevated risk of requiring reoperation after undergoing ACI, which is associated with an inability to attain clinically relevant improvements. Consequently, there is a compelling need for a thorough evaluation of the indications for ACI, particularly when patients have a history of previous surgeries. It is essential to ensure that both female patients and those with prior surgical interventions are well-informed about the potential for less favorable outcomes.

### Supplementary Information

Below is the link to the electronic supplementary material.

Supplementary file1 (DOCX 14 KB)

## Data Availability

The data that support the findings of this study are available from the corresponding author, AB, upon reasonable request.
