# Supplementary material for: Increased risk of reoperation and failure to attain clinically relevant improvement following autologous chondrocyte implantation of the knee in female patients and individuals with previous surgeries: a time-to-event analysis based on the German cartilage registry (KnorpelRegister DGOU)
Source: Knee Surg Sports Traumatol Arthrosc. 2023 Nov 11;31(12):5837–47. doi: 10.1007/s00167-023-07615-5 (PMC10719132; doi:10.1007/s00167-023-07615-5)
Supplement: Supplementary file 1 — Supplementary file1 (DOCX 14 KB) [file 167_2023_7615_MOESM1_ESM.docx]

| Table 4. Multicollinearity of Predictor Variables | | | |
| --- | --- | --- | --- |
|  | **Variable** | **VIF** | **R^2^ with other variables** |
| β1 | Age | 12.2 | 0.918 |
| β2 | Sex (Female) | 1.7 | 0.404 |
| β3 | BMI | 14.2 | 0.929 |
| β4 | Symptom Duration | 1.2 | 0.129 |
| β5 | Lesion Size | 4.6 | 0.783 |
| β6 | Lesion Localization (TF) | 1.9 | 0.477 |
| β7 | Previous Surgeries (1 - 2) | 2.1 | 0.530 |
| β8 | Previous Surgeries (> 2) | 1.2 | 0.173 |
| Multicollinearity of predictor variables in the cox proportional-hazards model for reoperation is demonstrated based on R^2^ and variance inflation factor (VIF). Tibio-femoral (TF), Body-Mass-Index (BMI) | | | |
